# Being Born Large for Gestational Age is Associated with Increased Global Placental DNA Methylation

**DOI:** 10.1038/s41598-020-57725-0

**Published:** 2020-01-22

**Authors:** S. E. Dwi Putra, C. Reichetzeder, A. A. Hasan, T. Slowinski, C. Chu, B. K. Krämer, B. Kleuser, B. Hocher

**Affiliations:** 1 Fifth Department of Medicine (Nephrology/Endocrinology/Rheumatology), University Medical Centre Mannheim, University of Heidelberg, Heidelberg, Germany; 2 Department of Nutritional Toxicology, Institute of Nutritional Science, University of Potsdam, Nuthetal, Germany; 3 Faculty of Biotechnology, University of Surabaya, Surabaya, Indonesia; 4 Department of Biochemistry, Faculty of Pharmacy, Zagazig University, Zagazig, Egypt; 5 UP Transfer GmbH, University of Potsdam, Potsdam, Germany; 6 Department of Nephrology, Campus Charité Mitte, University Hospital Charité, Berlin, Germany; 7 Department of Basic Medicine, Medical College of Hunan Normal University, Changsha, China; 8 Reproductive and Genetic Hospital of CITIC-Xiangya, Changsha, China

**Keywords:** Epidemiology, Molecular medicine

## Abstract

Being born small (SGA) or large for gestational age (LGA) is associated with adverse birth outcomes and metabolic diseases in later life of the offspring. It is known that aberrations in growth during gestation are related to altered placental function. Placental function is regulated by epigenetic mechanisms such as DNA methylation. Several studies in recent years have demonstrated associations between altered patterns of DNA methylation and adverse birth outcomes. However, larger studies that reliably investigated global DNA methylation are lacking. The aim of this study was to characterize global placental DNA methylation in relationship to size for gestational age. Global DNA methylation was assessed in 1023 placental samples by LC-MS/MS. LGA offspring displayed significantly higher global placental DNA methylation compared to appropriate for gestational age (AGA; p < 0.001). ANCOVA analyses adjusted for known factors impacting on DNA methylation demonstrated an independent association between placental global DNA methylation and LGA births (p < 0.001). Tertile stratification according to global placental DNA methylation levels revealed a significantly higher frequency of LGA births in the third tertile. Furthermore, a multiple logistic regression analysis corrected for known factors influencing birth weight highlighted an independent positive association between global placental DNA methylation and the frequency of LGA births (p = 0.001).

## Introduction

Birth size is an important clinical parameter that can be used for determining the health of the newborn and is highly correlated with postpartum morbidity and complications during labour. Increases in birth weight parallel the gestational age^1^. Relating birth weight to gestational age has the advantage of evaluating the potential growth of the fetus according to the length of pregnancy^2^. Weight for gestational age is usually classified into small for gestational age (SGA), appropriate for gestational age (AGA), and large for gestational age (LGA)^3^. Abnormal fetal growth is defined as being born smaller than the 10^th^ percentile (SGA) or larger than the 90^th^ percentile (LGA)^3^. Both SGA and LGA births display an increased peri- and postnatal morbidity and mortality. Regarding maternal health, SGA and LGA births are associated with maternal disease such as hypertension, preeclampsia, and preexisting or gestational diabetes mellitus. Moreover, SGA and especially LGA births are associated with adverse obstetrical outcomes such as emergency cesarean deliveries and postpartum hemorrhages^4^. SGA newborns are at an increased risk for stillbirth, seizures, intraventricular hemorrhages, hypoxic ischemic encephalopathy, necrotizing enterocolitis, sepsis, and neonatal mortality^5^. LGA newborns display an increased risk for stillbirth, traumatic delivery, brachial plexus palsy, mechanical ventilation, and neonatal mortality^5^. Furthermore, numerous studies have demonstrated that both SGA and LGA newborns are at an increased risk for disease in later life, highlighting the importance of these anthropometric measures as surrogate parameters of fetal programming^6–9^.

Adaptive processes of the fetus to adverse environmental cues during gestation can have an impact on the physiology and metabolism in later life^10,11^. An adverse intrauterine environment can be translated into being born small for gestational age (SGA) which is an independent risk factor for metabolic diseases^12,13^. Interestingly, similar associations have been demonstrated for LGA newborns, displaying an increased susceptibility for obesity, hypertension and diabetes mellitus compared to appropriate for gestational age (AGA) newborns^6,14^. Overall, studies show an U-shaped relation between birth weight and type-2 diabetes, hypertension or risk of overweight in later life^15,16^. However, it has been proposed that the underlying mechanisms leading to the same metabolic diseases in adult life differ between SGA and LGA newborns^17,18^.

The placenta is the primary means of communication between mother and fetus during pregnancy. Placental function has been reported to be altered in pregnancies with SGA or LGA outcomes^19^. Changes in placental function may occur due to changes in placental structure. However, the molecular pathways responsible for changes in placental structure and function in association with pregnancy outcomes have not been widely studied yet^19^. More recent studies highlight the importance of epigenetic mechanisms, primarily DNA methylation, in placental performance during pregnancy^20–22^.

Birth weight for gestational age is a complex phenotype that results from various processes and the expression of numerous genes in the genome. This might explain why association analyses between DNA methylation of certain genes and birth weight for gestational age remained elusive despite a direct connection between the gene of interest and fetal growth processes^23,24^. The whole human genome contains only about 1.5% of protein encoding genes, while the remaining majority comprises of introns, repetitive elements, and other non-coding sequences^25^. Initially regarded as “junk DNA” more recent research has revealed important functions of non-coding genomic regions and their epigenetic modification, including DNA methylation^26,27^. Thus, investigating DNA methylation levels on a global scale might facilitate getting first insights into epigenetic mechanisms of complex phenotypes^28,29^. However, studies investigating correlations between global DNA methylation and birth weight so far were only performed using rather small sample sizes^30–33^.

Currently, various approaches to quantify global DNA methylation exist. Several studies applied genome wide array approaches^34–36^. Although such assays can measure CpG island methylation of about 99% of all RefSeq genes, they only cover about 1.5% of genomic CpGs, are more specifically designed to analyze promotor methylation and neglect other genomic regions where DNA methylation might be of importance^37,38^. Also newer variants of genome wide array approaches like the Illumina MethylationEPIC bead chip still exclude regions of potentially meaningful biological variation^38^. More comprehensive methods such as whole genome bisulfite sequencing are not feasible yet for utilization with large sample sizes. Other novel sequencing based approaches that indeed are applicable for large sample sizes do so at the expense of genomic coverage and are further limited by their still quite extensive costs^38^. Other commonly used methods measure DNA methylation in repetitive elements such as LINE-1 or Alu elements that serve as surrogate parameters for global DNA methylation^39^. Futhermore, the absolute methyl cytosine content can be measured by using HPLC or LC-MS/MS. LC-MS/MS is reported to be the most sensitive method for absolute methyl cytosine quantification and is considered the current gold standard^37^.

The aim of this study was to analyze placental global DNA methylation using the current gold standard method, LC-MS/MS, and to investigate whether alterations in birth weight for gestational age are associated with differences in global placental DNA methylation.

## Material and Methods

### Clinical study

The study was conducted in 1023 mothers with singleton pregnancies delivering at the Charité obstetrics department, Berlin, Germany from 2000 to 2008. The local ethics committee approved the study and all clinical investigations were performed according to the principles expressed in the Declaration of Helsinki. Written informed consent was obtained from all participants. Data on history of diabetes in the family, the status of diabetes before or during pregnancy, smoking status before pregnancy and the ethnic background were obtained from the “Mutterpass” (German pregnancy record booklet). According to the practice guideline of the German Diabetes Assosiation (DGG) and the German Association for Gynaecology and Obstetrics (DGGG), all mothers were screened for gestational diabetes^40^. Variables describing diabetes in the dataset were diabetes before pregnancy (preexisting diabetes mellitus according to the pregnancy record booklet) and diabetes during pregnancy (composite of diabetes before pregnancy and gestational diabetes). Gestational age was estimated from the date of the last menstrual period. Data on blood pressure during pregnancy were obtained from routine antenatal examinations. Placental samples were collected directly after delivery. One complete placental cotyledon taken from the same location was collected from all participating mothers. Placental samples were stored at −20 °C until further use for DNA isolation. Additionally, data regarding the sex of the newborn, birth weight, birth length, and head circumference were extracted into our database.

### DNA preparation and placental methylation quantification

DNA extraction was performed using a QIAamp DNA Mini Kit (Qiagen). RNase was added during the isolation process to eliminate the influence of RNA in the next step. Quality and quantity of the DNA solution were measured using a spectrophotometer ND-1000 (NanoDrop). Degradase plus DNAse (Zymo Research) was used in the DNA digestion process according to the manufacturer’s instructions. As a control, 200 ng of the digested DNA was analyzed by agarose gel electrophoresis. DNA was prepared by adding 280 µl of 0.1% formic acid to 70 µL of digested DNA to obtain a final concentration of 2 ng/µL. Determination of placental methylation was performed by liquid chromatography-electrospray ionization/multi-stage mass spectrometry (LC-ESI/MS/MS) as described previously^41^. Analyses were performed with an Agilent 6530 Accurate-Mass Q-TOF instrument with Jet Stream-Interface (Palo Alto, USA). The chromatographic separation process was performed with a X-BridgeTM C18 4.6 mm × 150 mm (3.5 lm particle size) column (Waters). For gradient elution solvent A (0.1% formic acid in water) and solvent B (0.1% formic acid in methanol) were used. Flow rate of eluent was maintained constant at 0.5 ml/min. A total of 100 ng digested DNA in a volume of 50 µL was applied in every injection. The parameters used in the ESI-MS/MS step for gas temperature and gas flow rate were 250 °C and 8 L/min respectively, while the sheat nebulizer gas temperature and pressure were maintain at 300 °C and 60 psig. Parameters associated with the voltage were as follows: capillary voltage 4000 V; collision energy for dC, and dG 5mdC row 7 V, 13 V and 10 V. The quantification of placental methylation was calculated based on the formula: Methylation % = [5mdC]/{[5mdC] + [dC]} over m/z signal for dC and mdC at 228.0979/112.0505 and 242.1135/126.0662, respectively.

### Statistical analysis

SPSS version 20 was used for the statistical analyses. Descriptive data are given as mean plus/minus standard deviation for continuous variables or percent for dichotomous variables. SGA was defined as birth weight for gestational age below the 10th percentile, while the cutoff for LGA was above the 90th percentile, using a global reference. ^2^. For the comparison of means between groups, ANOVA was applied according to normal distribution of the data. A Bonferroni post hoc test was used to assess specific significant differences between two groups. Kruskal-Wallis test was applied for non-normally distributed data. Independence of observed associations was investigated using a multivariable ANCOVA model adjusted for suitable confounders. Parameters that were already shown to interact with global DNA methylation and/or variables that were significantly different among SGA, AGA, and LGA births were employed as confounders. For the ANCOVA model these included as diabetes during pregnancy, age of the mother, systolic blood pressure, and smoking before pregnancy^42–47^. Associations between DNA methylation stratified into tertiles and the frequency for AGA, SGA or LGA was analyzed using Pearson’s Chi-square test. Analysis of the independence of observed associations was performed employing a multiple logistic regression analysis. Variables known to impact on size for gestational age and/or variables that were significantly different among the three ranked global placental DNA methylation groups were included as confounders. These parameters consisted of diabetes during pregnancy, smoking before pregnancy, pre-pregnancy BMI, diastolic blood pressure, ethnicity, and sex of the child^48,49^. Collinearity between confounders was controlled by using a variance inflation factor <5^50^.

### Ethics approval

The study was approved by the ethical comitee of the University Hospital Charité, Berlin-Mitte, Humboldt-University Berlin, Berlin, Germany.

### Consent for publication

The final version of this manuscript was approved by all authors for submission to Scientific Reports

## Results

Detailed descriptive statistics of the cohort are shown in Table 1. The mean age of the mothers was 30.0 ± 5.9 years. The overall mean gestational age at birth, birth weight, and birth length index were 38.8 ± 2.0 weeks, 3355.4 ± 630.1 g, and 50.7 ± 3.2 cm, respectively. Regarding the distribution of sex, 52.3% were male and 47.7% were female newborns. Placental DNA methylation ranged from 2.01% to 4.83%, with a mean value of 3.01 ± 0.47%. Descriptive statistics of the cohort grouped according to the birth weight for gestational age are displayed in Table 1. There were 127 SGA, 796 AGA and 100 LGA newborns in this study. LGA newborns were heavier (p < 0.001), longer (p < 0.001), and had a bigger head circumference (p < 0.001) compared to AGA and SGA newborns. Gestational age and pre-pregnancy BMI were also significantly different (p = 0.002 and p < 0.001). The distribution of sex was significantly different (p < 0.001), with SGA births showing a higher frequency of female (34.6% males; 65.4% females) and LGA births a higher frequency of male newborns (69.0% males; 31.0% females). Moreover, the distribution of the mothers who suffered from diabetes mellitus during pregnancy was significantly shifted towards LGA newborns (p < 0.001).

**Table 1:** Descriptive statistics of all mother-child pairs and group comparison according to birth weight for gestational age; Data are given as mean ± SD or %; SGA = small for gestational age; AGA = appropriate for gestational age; LGA = large for gestational age; BMI = body mass index; SBP = systolic blood pressure; DBP = diastolic blood pressure.

Parameter	All samples	SGA	AGA	LGA	p
	(N = 1023)	(N = 127)	(N = 796)	(N = 100)	
Placental methylation (%)	3.01 ± 0.47	2.99 ± 0.41	2.99 ± 0.47	3.19 ± 0.54	<0.001
Age of the mother (years)	30.0 ± 5.9	29.6 ± 5.9	30.0 ± 5.9	31.0 ± 5.7	0.161
Pre-pregnancy BMI (kg/m2)	23.2 ± 4.6	22.2 ± 4.0	23.1 ± 4.5	25.1 ± 5.8	<0.001
SBP 3rd trimester (mmHg)	116.3 ± 11.2	114.8 ± 12.3	116.4 ± 10.9	117.6 ± 11.7	0.162
DBP 3rd trimester (mmHg)	70.5 ± 7.6	70.3 ± 8.9	70.5 ± 7.4	70.3 ± 7.5	0.901
Smoking before pregnancy (%)	36.3	41.3	35.9	33.0	0.393
Diabetes before pregnancy (%)	1.0	0.8	0.8	3.0	0.092
Diabetes during pregnancy (%)	6.3	7.1	4.9	16.0	0.001
Diabetes in family (%)	36.6	43.4	35.5	36.2	0.312
Ethnicity (Caucasian/other; %)	93.3/6.7	89.8/10.1	93.6/6.4	95.0/5.0	0.214
Gestational age at delivery (weeks)	38.8 ± 2.0	38.2 ± 2.3	38.9 ± 2.0	38.7 ± 2.2	0.002
Child birth weight (g)	3355.4 ± 630.1	2515.2 ± 355.3	3380.0 ± 493.1	4226.9 ± 554.4	<0.001
Child head circumference (cm)	34.7 ± 1.7	32.9 ± 1.5	34.8 ± 1.5	36.3 ± 1.3	<0.001
Child birth length (cm)	50.7 ± 3.2	47.3 ± 3.1	50.9 ± 2.8	53.5 ± 2.8	<0.001
Sex of the child (m/f; %)	52.3/47.7	34.6/65.4	53.0/47.0	69.0/31.0	<0.001

**Figure 1:**
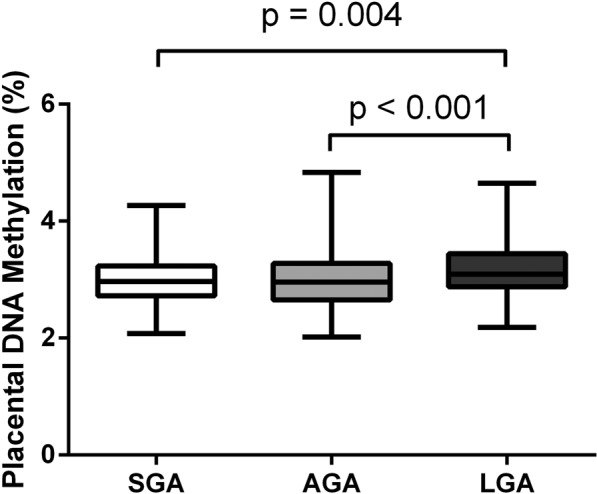
ANOVA analysis followed by a Bonferroni post-hoc test comparing global placental DNA methylation among birth weight for gestational age groups. Box and whisker blot showing median, minimum and maximum; SGA = small for gestational age; AGA = appropriate for gestational age; LGA = large for gestational age.

To investigate potential differences in global placental DNA methylation between the three birthweight for gestational age groups, ANOVA analyses followed by a Bonferroni post hoc test were performed. These analyses revealed a significantly higher global placental DNA methylation in LGA compared to AGA (p < 0.001) and SGA (p = 0.004) newborns (Fig. 1). There was no significant difference in global placental DNA methylation between AGA and SGA newborns as shown in Fig. 1. To analyse the potential impact of various confounders on global placental DNA methylation, a multivariable ANCOVA model was calculated including diabetes during pregnancy, age of the mother, systolic blood pressure and smoking before pregnancy (Table 2). This analysis demonstrated that higher levels of global placental DNA methylation are associated with LGA births independent of other confounding factors known to influence DNA methylation (p < 0.001; Partial η^2^ = 0.013; 95% C.I. = 0.08–0.28; Table 2).

**Table 2:** ANCOVA analysis to investigate the association between global placental DNA methylation (dependent variable) and birth weight for gestational age (^a^AGA was used as a reference); SGA = small for gestational age; AGA = appropriate for gestational age; LGA = large for gestational age; BMI = body mass index; SBP = systolic blood pressure;.

Dependent variable: Placental DNA methylation; r^2^ = 0.026	B	S.E.	Partial η2	p	95% C.I. for B	
					Min.	Max.
Intercept	3.29	0.17	0.262	<0.001	2.95	3.63
Pre-pregnancy BMI (kg/m^2^)	0.00	0.00	0.000	0.487	−0.01	0.00
Diabetes during pregnancy (yes/no)	0.19	0.06	0.009	0.003	0.07	0.31
Smoking before pregnancy (yes/no)	−0.06	0.03	0.002	0.038	−0.13	0.00
SBP (mmHg)	0.00	0.00	0.004	0.157	0.00	0.00
Age of the mother (years)	0.00	0.00	0.000	0.966	0.00	0.01
SGA	0.00	0.05	0.000	0.963	−0.09	0.09
LGA	0.18	0.05	0.013	<0.001	0.08	0.28
AGA	0^a^					

To further investigate the association between LGA births and increased global placental DNA methylation, tertiles of global placental DNA methylation were created and ranked into low (n = 341), moderate (n = 341), and high (n = 341) DNA methylation. Descriptive statistics of mothers ranked into tertiles according to the degree of global placental DNA methylation are shown in Table 3. Several parameters were significantly different between the three groups, including diastolic blood pressure (p = 0.017), ethnicity (p = 0.030) smoking before pregnancy (p = 0.005) and diabetes during pregnancy (p = 0.006). Moreover, Pearson’s Chi-square test was performed to assess the prevalence of SGA, AGA and LGA births among the three placental DNA methylation groups (Table 4). There was a significant difference (Pearson’s Chi-Square: 19.051; p = 0.001) in the prevalence for SGA, AGA, and LGA births. Mothers in the group of highest global placental DNA also displayed the highest prevalence for LGA offspring (48.0% (LGA) vs. 32.4% (AGA) vs. 27.6% (SGA); Table 4). Moreover, to investigate a potential independent association between birth weight for gestational age and global placental DNA methylation, an adjusted multiple logistic regression model was calculated using birth weight for gestational age as dependent and global placental DNA methylation as independent continuous variable. The model was adjusted for important factors impacting on size for gestational age and variables that were significantly different among three groups of ranked global placental DNA methylation. These included diabetes during pregnancy, pre-pregnancy BMI, ethnicity, sex of the child, diastolic blood pressure and smoking before pregnancy. The multiple logistic regression model highlighted an independent positive association between LGA birth and global placental DNA methylation (p = 0.001; Exp(B) = 2.06; 95% C.I. = 1.35–3.16; Table 5).

**Table 3:** Descriptive statistics of mother-child pairs stratified into tertiles of global placental DNA methylation (Low; Moderate; High); Data are given as mean ± SD or %; BMI = body mass index; SBP = systolic blood pressure; DBP = diastolic blood pressure.

Parameter	Methylation level			p
	Low	Moderate	High	
Placental DNA methylation (%)	2.52 ± 0.18	2.98 ± 0.11	3.53 ± 0.34	<0.001
Age of the mother (years)	29.7 ± 6.0	30.1 ± 6.2	30.3 ± 5.6	0.382
Pre-pregnancy BMI (kg/m^2^)	23.3 ± 4.6	23.2 ± 4.4	23.1 ± 4.8	0.819
SBP 3rd trimester (mmHg)	116.9 ± 10.9	116.7 ± 11.3	115.2 ± 11.3	0.089
DBP 3rd trimester (mmHg)	71.4 ± 7.5	70.1 ± 7.7	69.9 ± 7.4	0.017
Smoking before pregnancy (%)	43.2	33.5	32.2	0.005
Diabetes before pregnancy (%)	0.6	1.2	1.2	0.666
Diabetes during pregnancy (%)	4.4	4.7	9.7	0.006
Diabetes in family (%)	37.7	35.3	36.6	0.854
Ethnicity (Caucasian/other; %)	94.7/5.3	94.7/5.3	90.3/9.7	0.030
Gestational age at delivery (weeks)	38.8 ± 2.1	38.7 ± 2.1	38.8 ± 2.0	0.573
Child birth weight (g)	3367.0 ± 585.9	3295.9 ± 643.8	3403.4 ± 655.4	0.077
Child head circumference (cm)	34.8 ± 1.6	34.6 ± 1.8	34.8 ± 1.7	0.557
Child birth length (cm)	50.8 ± 2.9	50.4 ± 3.3	50.9 ± 3.3	0.066
Sex of the child (m/f; %)	50.7/49.3	48.1/51.9	44.3/55.7	0.238

**Table 4:** Cross-tabulation of global placental DNA methylation ranked in tertiles and the frequency of different birth weight for gestational age groups (SGA; AGA; LGA); SGA = small for gestational age; AGA = appropriate for gestational age; LGA = large for gestational age.

Pearson’s Chi-Square: 19.051; p = 0.001		Placental DNA methylation ranked		
		Low	Moderate	High
SGA	Count	36	56	35
	% within placental DNA methylation rank	28.3	44.1	27.6
AGA	Count	283	255	258
	% within placental DNA methylation rank	35.6	32.0	32.4
LGA	Count	22	30	48
	% within placental DNA methylation rank	22.0	30.0	48.0
Total	Count	341	341	341

**Table 5:** Multiple logistic regression analysis of the association between global placental DNA methylation and birth weight for gestational age (dependent variable); ^a^AGA was set as reference for this parameter; AGA = appropriate for gestational age; LGA = large for gestational age; BMI = body mass index; DBP = diastolic blood pressure.

Parameter		B	S.E	p	Exp(B)	95% C.I. for Exp(B)	
						Min.	Max.
**LGA**^**a**^	Intercept	−4.39	1.38	0.001			
	Diabetes during pregnancy (yes/no)	1.05	0.34	0.002	2.86	1.47	5.57
	Smoking before pregnancy (yes/no)	−0.16	0.24	0.507	0.85	0.54	1.36
	BMI beginning of pregnancy (kg/m^2^)	0.08	0.02	<0.001	1.08	1.04	1.13
	DBP (mmHg)	−0.02	0.02	0.230	0.98	0.95	1.01
	Ethnicity (Caucasian, other)	0.40	0.51	0.430	1.49	0.55	4.01
	Sex of the child (male/female)	−0.65	0.23	0.005	0.52	0.33	0.83
	Placental methylation	0.72	0.22	0.001	2.06	1.35	3.16

## Discussion

The current study demonstrated that being born LGA is associated with a significantly higher level of global placental DNA methylation compared to AGA or SGA births. An ANCOVA model furthermore demonstrated that this association is independent of known confounders of placental DNA methylation (p < 0.001; Partial η^2^ = 0.013; 95% C.I. = 0.08–0.28). To substantiate the finding of an association between LGA and higher global placental DNA methylation, global placental DNA methylation was ranked into three groups comprising of low, moderate and high DNA methylation. In these groups, the frequency of LGA births was compared. These analyses demonstrated a significantly higher frequency of LGA births in the group of mothers with the highest global placental DNA methylation levels. Furthermore, by comparing descriptive statistics of the three methylation groups, potential confounders of the association between higher global placental DNA methylation and LGA births were identified. Diastolic blood pressure, ethnicity, the prevalence of smoking before pregnancy, and the prevalence of diabetes were significantly different among the three methylation groups. Calculating a multiple logistic regression model adjusted for the identified confounders and other well-established confounders of birth weight for gestational age plus using placental methylation as a continuous variable backed results of the univariate analysis, highlighting an independent positive association between global placental DNA methylation and LGA births (p = 0.001; Exp(B) = 2.06; 95% C.I. = 1.35–3.16).

The average global placental methylation assessed by this study was 2.99 ± 0.47% in AGA newborns. This finding is in agreement with previous studies, demonstrating global hypomethylation in comparison to other tissues in the human body^51^. However, different to previous studies which used HPLC to quantify global placental DNA methylation, this study employed the current gold standard of global DNA methylation quantification, LC-MS/MS, and is by far the largest study of its kind.

In a previous study we observed an association between gestational diabetes mellitus and higher levels of global placental DNA methylation in a similar cohort as the one analysed in this study^47^. As to be expected, ranking global placental DNA methylation into 3 groups demonstrated a higher frequency of diabetes during pregnancy (composite variable encompassing mothers with overt diabetes before and during pregnancy) in the highest global placental DNA methylation tertile. As diabetes during pregnancy is strongly associated with an increased risk for LGA births and, as previously shown, is associated with increased global placental DNA methylation, any multivariable analysis had to be adjusted for maternal diabetes. However, adjusting for diabetes during pregnancy in the multivariable logistic regression model did not affect the association between increased levels of global placental DNA methylation and a higher frequency for LGA births. Furthermore, an evaluation of the study cohort excluding any mothers with diabetes (Supplemental Data) did not weaken but actually slightly strengthen the association between global placental DNA methylation and LGA births. This suggests that the association between gestational diabetes and global placental DNA methylation is an independent phenomenon and does not explain the observed association between global placental DNA methylation and LGA birth.

Several studies have been conducted to investigate the impact of global placental DNA methylation on birth weight. Most of these studies measured LINE-1 methylation as a surrogate for global DNA methylation, were performed in rather small scale settings, and were more focused on intrauterine growth restriction (IUGR) or SGA birth outcomes^30–33^. Bourque *et al*., using 35 placenta samples, showed no difference in LINE-1 methylation comparing intrauterine growth restricted newborns to newborns with normal birth weight^30^. Tzschoppe *et al*., measuring LINE-1 methylation in 55 placenta samples showed no difference in placental DNA methylation between IUGR, SGA and AGA newborns^32^. Similarly, Mukhopadhyay *et al*. did not observe significant associations between placental LINE-1 methylation and birth weight analyzing 109 placenta samples of AGA and SGA births^31^. Different to just mentioned studies, Xiao *et al*., using 119 placenta samples, revealed a positive correlation between placental LINE-1 methylation and birth weight standard deviation scores^33^. Michels *et al*. who investigated LINE-1 methylation in 319 placenta samples observed a higher degree of placental methylation in the low birth weight group (<2500 g) compared to the normal birth weight group (2500–4000 g)^52^. Contrary to these results, we did not observe any significant differences in global placental DNA methylation of SGA newborns. Similar to findings of the current study, Wilhelm-Benartzi *et al*., analyzing DNA methylation of repetitive elements in 380 placenta samples, showed that placental methylation of the Alu element AluYB8 was significantly higher in LGA offspring^53^. Furthermore, both LINE-1 and Alu methylation levels were significantly positively associated with birth weight percentiles^53^. Overall, the rather heterogeneous supporting and opposing findings of previous studies can only be carefully used for interpreting our current findings. Major difference of the current study is the usage of a methodology that robustly quantifies global DNA methylation, in contrast to the usage of surrogate parameters of global DNA methylation in previous studies^30,32,33,52,53^. Furthermore, chosen sample size appears to be an important factor, given the differences in results comparing small scale^30–32^ to relatively larger scale studies^52,53^.

The potential biological effect of increased global placental DNA methylation in general and even more so in regards to affecting growth patterns of the offspring remains very poorly understood. The placenta does share certain phenotypic features usually found in solid neoplasms, such as cell invasion, vascular remodelling and suppression of the immune system^54^. Moreover, the placenta, similar to many neoplasms, is characterized by global DNA hypomethylation in comparison to somatic tissues^55^. Interestingly, next to global hypomethylation, neoplastic tumors demonstrate local hypermethylation, which is often found in tumor suppressor genes^56,57^. Hypermethylation of tumor suppressor genes was also demonstrated for the human placenta^58^. Moreover, as outlined above, increases in methylation of Alu elements, was shown to be positively correlated with fetal growth^53^. Higher methylation in these evolutionary younger transposons, was found to be relevant for placental function^59^. Furthermore, it was demonstrated in a study in rats that the administration of the DNA methylation inhibitor 5-Azacitidine lead to significantly smaller placentas, yet again highlighting a connection between the degree of placental DNA methylation and placental growth. Placental weight, which serves as a surrogate parameter of the surface area for materno-fetal nutrient transport, is a very important determinant for fetal growth and offspring birth weight^60,61^. Hypothetically, above average hypermethylation of growth related regions could induce larger than average placental growth which could translate into an increased risk for LGA births. Furthermore, above average increases in DNA methylation of diverse growth related regions in the placental DNA could result in a slight net increase in global placental DNA methylation that differentiates LGA from AGA births. However, to address these issues future adequately designed studies are necessary before drawing any causal conclusions.

Findings of the current study can be interpreted by findings of studies investigating other aspects of global placental DNA methylation. Nomura *et al*. showed that obese women display a higher global placental DNA methylation compared to lean women^62^. It is well established that obesity is a strong maternal risk factor for LGA offspring^6^. In the current study we did not observe any association between pre-pregnancy BMI and global placental DNA methylation. However, different from the study of Nomura *et al*. in which obesity was defined as a BMI > 30 kg/m^2^ and affected 30% of the participating mothers, mean pre-pregnancy BMI in the current study was 23.2 ± 4.6 kg/m^2^. It is possible that these differences in the makeup of the investigated cohorts accounted for the conflicting observation. Although increases in BMI are associated with dyslipidemia^63^, studies demonstrated BMI independent associations between the maternal blood lipid profile and LGA births^64–66^. Global DNA methylation has been reported to be associated with lipid metabolism and plasma glucose concentration^67^. It was demonstrated in a rat model that maternal dyslipidemia, induced by feeding a high fat diet, increases global placental DNA methylation^68^. Interestingly, an intervention with dietary n-3 fatty acids reversed these alterations^68^. Current literature suggests that the link between dietary lipids and DNA methylation might be due to their involvement in one carbon metabolism^68,69^. Thus, the maternal genetic setup or consumed diet^70^, key factors that affect blood lipid profiles, might also impact on placental lipid metabolism and DNA methylation during pregnancy. Effects of changes in the placental DNA methylation status on placental lipid metabolism or, vice versa, effects of an altered placental lipid metabolism on the placental DNA methylation status theoretically could be one factor for the positive association between DNA methylation and LGA offspring and warrant further studying.

### Study strengths and limitations

One limitation of this and the majority of previous studies is that no cell type separation prior to DNA methylation analyses was performed. The placenta is composed of several different cell types ranging from fibroblast, trophoblasts to mesenchymal cells. Epigenetic patterns are cell specific and different cell types in the placenta are known to have a different DNA methylation profile^71^. Thus, it is not possible to rule out that the obtained results were influenced by cell type heterogeneity in between samples. However, since this study used more than 1000 placenta samples, isolation and analysis of specific placental cells was not feasible. There are studies that compared the effect of different placental sampling areas and the isolation of specific placental cell types on global placental DNA methylation^72,73^. Non *et al*. showed that sampling from different locations in the human placenta still resulted in strongly correlated degrees of LINE-1 methylation^73^. Schroeder *et al*. compared global DNA methylation, measured by MethylC-Seq in isolated rhesus trophoblasts to whole rhesus placenta methylation by MethylC-Seq and demonstrated a strong correlation between the two^72^. However, studies that focus on elucidating the contribution of individual cell types to whole placental DNA methylation are still lacking. This is especially problematic for the analysis of array data, as until now accounting for mixed cell type composition, an important consideration in heterogeneous tissues such as the placenta, is still not feasible^74^. Thus, future studies that focus on cell type specific differences in placental DNA methylation and their contribution to whole placenta DNA methylation are urgently needed.

Next to the mentioned limitations, this study has several strengths. To the best of our knowledge, it is by far the largest study of its type, investigating global placental DNA methylation in over a thousand placenta samples. More so, the current gold standard for the quantification of global DNA methylation, LC-MS/MS, was used^75^.

## Conclusions

The current study demonstrated a significant positive association between the degree of global placental DNA methylation and being born LGA, which was independent of maternal diabetes during pregnancy, a very important risk factor for LGA births. Taken together, evidence in literature is existing that supports the observed positive association between global placental DNA methylation and being born LGA. So far the underlying mechanisms remain elusive. Hypothetically, hypermethylation of growth related DNA regions, such as tumor suppressor genes and certain transposable elements could affect placental size and function which could impact on fetal growth. Above average increases of DNA methylation in growth related DNA regions could result in pathologically increased growth. Furthermore, this might be reflected by significantly elevated levels of global placental DNA methylation. Findings of previous studies suggest a possible connection between anthropometric measurements, lipid metabolism, one-carbon metabolism, and DNA methylation. Taken together, it remains largely unknown how changes in global placental DNA methylation can affect fetal growth. Moreover, the cause of altered global placental DNA methylation levels in LGA births is still highly elusive. Thus, future studies are needed to be able to draw any more causal conclusions.

## Supplementary information

Supplementary Dataset 1.

## Data Availability

Data will be made available upon request to the corresponding author
