# Supplementary material for: Being Born Large for Gestational Age is Associated with Increased Global Placental DNA Methylation
Source: Sci Rep. 2020 Jan 22;10:927. doi: 10.1038/s41598-020-57725-0 (PMC6976643; doi:10.1038/s41598-020-57725-0)
Supplement: Supplementary file 1 — Supplementary Dataset 1. [file 41598_2020_57725_MOESM1_ESM.docx]

# Being Born Large for Gestational Age is Associated with Increased Global Placental DNA Methylation

Dwi Putra SE^a,b,c,j^, [Reichetzeder C](http://www.ncbi.nlm.nih.gov/pubmed?term=Reichetzeder%20C%5BAuthor%5D&cauthor=true&cauthor_uid=24713853)^b,j,*^, Hasan AA^a,d.e^, Slowinski T^f^, Chu C^a^, Krämer BK , [Kleuser B](http://www.ncbi.nlm.nih.gov/pubmed?term=Kleuser%20B%5BAuthor%5D&cauthor=true&cauthor_uid=24713853)^b^, and [Hocher B](http://www.ncbi.nlm.nih.gov/pubmed?term=Hocher%20B%5BAuthor%5D&cauthor=true&cauthor_uid=24713853)^a,g,h,I,*^

1. Fifth Department of Medicine (Nephrology/Endocrinology/Rheumatology), University Medical Centre Mannheim, University of Heidelberg, Heidelberg, Germany
2. Department of Nutritional Toxicology, Institute of Nutritional Science, University of Potsdam, Nuthetal, Germany;
3. Faculty of Biotechnology, University of Surabaya, Surabaya, Indonesia;
4. Department of Biochemistry, Faculty of Pharmacy, Zagazig University, Zagazig, Egypt
5. UP Transfer GmbH, University of Potsdam, Potsdam, Germany;
6. Department of Nephrology, Campus Charité Mitte, University Hospital Charité, Berlin, Germany;
7. Department of Basic Medicine, Medical College of Hunan Normal University, Changsha, China;
8. LADR GmbH Neuruppin MVZ, Neuruppin, Germany
9. Reproductive and Genetic Hospital of CITIC-Xiangya, Changsha, China.
10. Equally contributing first authors;

***Abbreviated title:*** LGA birth and global placental DNA methylation

* Corresponding authors:

**Prof. Dr. Berthold Hocher**

University of Heidelberg

University Medical Centre Mannheim

Institute of Nutritional Science

Theodor-Kutzer-Ufer 1-3
68167 Mannheim, Germany

Telephone [0621/383-5172](http://w2.umm.de/tel:%2006213832663)
Telefax 0621/383-3804

E-Mail: [berthold.hocher@medma.uni-heidelberg.de](javascript:linkTo_UnCryptMailto('jxfiql7yboqelia+elzeboXjbajx+rkf:ebfabiybod+ab');)

**Dr. med. Christoph Reichetzeder**

University of Potsdam,

Institute of Nutritional Science,

Department of Nutritional Toxicology,

Arthur Scheunert Allee 114-116

14558 Nuthetal, Germany

E-Mail: reichetz@uni-potsdam.de

**Supplemental Data**

To investigate if the association between higher global placental DNA methylation and an increased frequency of LGA births is truly independent of diabetes during pregnancy all 64 mothers with pre-existing or gestational diabetes (variable: “diabetes during pregnancy”) were removed from the cohort. All descriptive statistics, the crosstabulation and the two multivariable models were recalculated. Regarding descriptive statistics of the cohort stratified into SGA, AGA, and LGA births no major changes were apparent. One noticeable but expected change was a slight shift in the respective birth weights which was most pronounced in the LGA group (SGA: 2515.2 ± 355.3 g (inclusive diabetic mothers) vs. 2520.0 ± 359.5 g (exclusive diabetic mothers); AGA: 3380.0 ± 493.1 g (inclusive diabetic mothers) vs. 3378.8 ± 492.3 g (exclusive diabetic mothers); LGA: 4226.9 ± 554.4 g (inclusive diabetic mothers) vs. 4195.7 ± 570.4 g (exclusive diabetic mothers). Removing mothers with diabetes during pregnancy did not affect the result of the ANCOVA analysis. LGA birth was still significantly and independently associated with global placental DNA methylation (p<0.001; Partial η^2^=0.015; 95% C.I.=0.010-0.31). The association was actually slightly stronger than in the ANCOVA analysis that included mothers with diabetes during pregnancy (p<0.001; Partial η^2^= 0.013; 95% C.I.= 0.08-0.28). Similar results were obtained for the multivariable logistic regression model. Also here the exclusion of mothers with diabetes during pregnancy did not affect the significant association between global placental DNA methylation and LGA births (p<0.001; Exp(B)=2.37; 95% C.I.= 1.47-3.80). Again, the association was more a bit pronounced than in the analysis that included mothers with diabetes during pregnancy (p=0.001; Exp(B)=2.06; 95% C.I.= 1.35-3.16).

*Supplemental Figure 1:* *ANOVA analysis followed by a Bonferroni post-hoc test comparing global placental DNA methylation among birth weight for gestational age groups. Graphs represent mean ± SEM; SGA = small for gestational age; AGA = appropriate for gestational age; LGA = large for gestational age;*

*Supplemental Table 1. Descriptive statistics of all mother-child pairs and group comparison according to birth weight for gestational age. Data are given as mean ± SD or %.*

| **Parameter** | **All samples** | **SGA** | **AGA** | **LGA** | **p** |
| --- | --- | --- | --- | --- | --- |
|  | **(N=959)** | **(N=118)** | **(N=757)** | **(N=84)** |  |
| Placental methylation (%) | 2.99 ± 0.46 | 2.97 ± 0.4 | 2.98 ± 0.45 | 3.18 ± 0.53 | 0.001 |
| Age of the mother (years) | 29.9 ± 5.9 | 29.5 ± 5.8 | 29.8 ± 5.9 | 31.0 ± 5.8 | 0.166 |
| Pre-pregnancy BMI (kg/m^2^) | 23.1 ± 4.4 | 22.1 ± 4.0 | 23.0 ± 4.2 | 25.2 ± 5.9 | <0.001 |
| SBP 3rd trimester (mmHg) | 116.4 ± 11.2 | 114.5 ± 11.8 | 116.4 ± 10.9 | 118.3 ± 11.9 | 0.053 |
| DBP 3rd trimester (mmHg) | 70.5 ± 7.5 | 70.1 ± 8.6 | 70.6 ± 7.4 | 70.6 ± 7.6 | 0.798 |
| Smoking before pregnancy (%) | 36.3 | 41.0 | 36.0 | 32.1 | 0.411 |
| Diabetes in family (%) | 35.9 | 43.9 | 34.9 | 33.8 | 0.231 |
| Ethnicity (Caucasian/other; %) | 895/64 | 107/11 | 708/49 | 80/4 | 0.393 |
| Gestational age at delivery (weeks) | 38.8 ± 2.0 | 38.2 ± 2.3 | 38.9 ± 2.0 | 38.7 ± 2.3 | 0.002 |
| Child birth weight (g) | 3344.7 ± 619.2 | 2520.0 ± 359.5 | 3378.8 ± 492.3 | 4195.7 ± 570.4 | <0.001 |
| Child head circumference (cm) | 34.7 ± 1.7 | 33.0 ± 1.5 | 34.9 ± 1.5 | 36.3 ± 1.3 | <0.001 |
| Child birth length (cm) | 50.7 ± 3.1 | 47.5 ± 2.9 | 50.9 ± 2.7 | 53.3 ± 3.0 | <0.001 |
| Sex of the child (m/f) | 500/459 | 42/76 | 400/357 | 58/26 | 0.001 |

BMI = body mass index; SBP = systolic blood pressure; DBP = Diastolic blood pressure;

*Supplemental Table 2. ANCOVA analysis to investigate the association between placental methylation (dependent variable) and birth weight for gestational age (^a^AGA was used as a reference)*

| **Dependent variable: Placental methylation; r^2^=0.019** | **B** | **S.E.** | **Partial**  **η^2^** | **p** | **95% C.I. for B** | |
| --- | --- | --- | --- | --- | --- | --- |
|  |  |  |  |  |  |  |
|  |  |  |  |  | Min. | Max. |
| Intercept | 3.37 | 0.18 | 0.278 | <0.001 | 3.02 | 3.72 |
| Pre-pregnancy BMI ( kg/m^2^) | -0.00 | 0.00 | 0.000 | 0.747 | -0.01 | 0.01 |
| Smoking before pregnancy (yes/no) | -0.07 | 0.03 | 0.005 | 0.027 | -0.13 | -0.01 |
| SBP (mmHg) | -0.00 | 0.00 | 0.004 | 0.061 | -0.01 | 0.00 |
| Age of the mother (years) | -0.00 | 0.00 | 0.000 | 0.684 | -0.01 | 0.00 |
| SGA | -0.00 | 0.05 | 0.000 | 0.967 | -0.09 | 0.09 |
| LGA | 0.20 | 0.05 | 0.015 | <0.001 | 0.10 | 0.31 |
| AGA | 0^a^ |  |  |  |  |  |

SGA = small for gestational age; AGA = appropriate for gestational age; LGA = large for gestational age; SBP = systolic blood pressure;

*Supplemental Table 3. Descriptive statistics of mother-child pairs stratified into tertiles of placental methylation (Low; Moderate; High). Data are given as mean ± SD or %.*

| **Parameter** | **Methylation level** | | | **p** |
| --- | --- | --- | --- | --- |
|  | **Low** | **Moderate** | **High** |  |
| Placental methylation (%) | 2.52 ± 0.18 | 2.97 ± 0.11 | 3.50 ± 0.33 | <0.001 |
| Gestational age at delivery (weeks) | 38.8 ± 2.0 | 38.7 ± 2.1 | 38.9 ± 2.0 | 0.599 |
| Age of the mother (years) | 29.6 ± 6.0 | 30.0 ± 6.1 | 30.0 ± 5.6 | 0.646 |
| Pre-pregnancy BMI (kg/m^2^) | 23.2 ± 4.7 | 23.1 ± 4.2 | 22.9 ± 4.4 | 0.595 |
| SBP 3rd trimester (mmHg) | 117.1 ± 10.9 | 116.8 ± 11.5 | 115.2 ± 11.1 | 0.067 |
| DBP 3rd trimester (mmHg) | 71.5 ± 7.5 | 70.1 ± 7.8 | 69.9 ± 7.2 | 0.014 |
| Smoking before pregnancy (%) | 43.1 | 33.5 | 32.4 | 0.009 |
| Diabetes in family (%) | 37.6 | 34.2 | 35.8 | 0.754 |
| Ethnicity (Caucasian/other; %) | 301/18 | 307/13 | 287/33 | 0.004 |
| Child birth weight (g) | 3356.3 ± 574.0 | 3293.8 ± 629.4 | 3384.0 ± 650.1 | 0.168 |
| Child head circumference (cm) | 34.8 ± 1.5 | 34.7 ± 1.7 | 34.7 ± 1.8 | 0.969 |
| Child birth length (cm) | 50.8 ± 2.8 | 50.4 ± 3.3 | 51.0 ± 3.0 | 0.052 |
| APGAR 5 min | 9.3 ± 1.1 | 9.4 ± 0.9 | 9.1 ± 1.0 | 0.015 |
| APGAR 10 min | 9.6 ± 0.9 | 9.7 ± 0.6 | 9.5 ± 0.8 | 0.045 |
| Sex of the child (m/f) | 158/161 | 167/153 | 175/145 | 0.427 |

BMI = body mass index; SBP = systolic blood pressure; DBP = Diastolic blood pressure;

*Supplemental Table 4. Cross-tabulation of placental methylation ranked in tertiles and birth weight for gestational age*

| **Pearson Chi-Square: 16.432; p=0.002** | | **Methylation ranked** | | |
| --- | --- | --- | --- | --- |
|  |  | **Low** | **Moderate** | **High** |
| **SGA** | Count | 34 | 52 | 32 |
|  | % within placental methylation rank | 28.8 | 44.1 | 27.1 |
| **AGA** | Count | 268 | 240 | 249 |
|  | % within placental methylation rank | 35.4 | 31.7 | 32.9 |
| **LGA** | Count | 17 | 28 | 39 |
|  | % within placental methylation rank | 20.2 | 33.3 | 46.4 |
| Total | Count | 319 | 320 | 320 |

SGA = small for gestational age; AGA = appropriate for gestational age; LGA = large for gestational age;

*Supplemental Table 5. Multiple logistic regression analysis of the association between placental methylation and birth weight for gestational age (dependent variable).*

| **Parameter** | | **B** | **S.E** | **p** | **Exp(B)** | **95% C.I. for Exp(B)** | |
| --- | --- | --- | --- | --- | --- | --- | --- |
|  |  |  |  |  |  | **Min.** | **Max.** |
| **LGA**^a^ | Intercept | -5.3 | 1.5 | 0.001 |  |  |  |
|  | Smoking before pregnancy (yes/no) | -0.2 | 0.3 | 0.418 | 0.81 | 0.49 | 1.35 |
|  | BMI beginning of pregnancy (kg/m^2^) | 0.1 | 0 | <0.001 | 1.10 | 1.05 | 1.15 |
|  | DBP (mmHg) | 0 | 0 | 0.270 | 0.98 | 0.95 | 1.01 |
|  | Ethnicity (Caucasian. other) | 0.41 | 0.6 | 0.463 | 1.51 | 0.50 | 4.53 |
|  | Sex of the child (male/female) | -0.6 | 0.3 | 0.011 | 0.53 | 0.32 | 0.87 |
|  | Placental methylation | 0.86 | 0.2 | <0.001 | 2.37 | 1.47 | 3.80 |

^a^ AGA was set as reference for these parameters; AGA = appropriate for gestational age; LGA = large for gestational age; BMI = body mass index. DBP = diastolic blood pressure;
